# Design rules for high mobility xanthene-based hole transport materials†
†Electronic supplementary information (ESI) available. See DOI: 10.1039/c9sc01491h


**DOI:** 10.1039/c9sc01491h

**Published:** 2019-07-25

**Authors:** Daniel P. Tabor, Valerie A. Chiykowski, Pascal Friederich, Yang Cao, David J. Dvorak, Curtis P. Berlinguette, Alán Aspuru-Guzik

**Affiliations:** a Department of Chemistry and Chemical Biology , Harvard University , 12 Oxford St. , Cambridge , MA 02138 , USA . Email: alan@aspuru.com; b Department of Chemistry , University of British Columbia , 2036 Main Mall , Vancouver , BC V6Y 1Z1 , Canada . Email: cberling@chem.ubc.ca; c Department of Chemistry , University of Toronto , 80 St. George Street , Toronto , ON M5S 3H6 , Canada; d Institute of Nanotechnology , Karlsruhe Institute of Technology , Hermann-von-Helmholtz-Platz 1 , 76344 Eggenstein-Leopoldshafen , Germany; e Stewart Blusson Quantum Matter Institute , The University of British Columbia , 2355 East Mall , Vancouver , BC V6T 1Z4 , Canada; f Department of Chemical and Biological Engineering , The University of British Columbia , 2360 East Mall , Vancouver , BC V6Y 1Z3 , Canada; g Department of Computer Science , University of Toronto , 214 College St , Toronto , ON M5T 3A1 , Canada; h Vector Institute , 661 University Ave Suite 710 , Toronto , ON M5G 1M1 , Canada

## Abstract

Tunable and highly conductive hole transport materials are crucial for the performance of organic electronics applications such as organic light emitting diodes and perovskite solar cells. For commercial applications, these materials' requirements include easy synthesis, high hole mobility, and highly tuned and compatible electronic energy levels. Here, we present a systematic study of a recently discovered, easy-to-synthesize class of spiro[fluorene-9,9′-xanthene]-based organic hole transport materials. Systematic side group functionalization allows us to control the HOMO energy and charge carrier mobility. Analysis of the bulk simulations enables us to derive design rules for mobility enhancement. We show that larger functional groups (*e.g.* methyl) decrease the conformational disorder due to steric effects and thus increase the hole mobility. Highly asymmetric or polar side groups (*e.g.* fluorine), however, increase the electrostatic disorder and thus reduce the hole mobility. These generally applicable design rules will help in the future to further optimize organic hole transport materials.

## Abstract 2

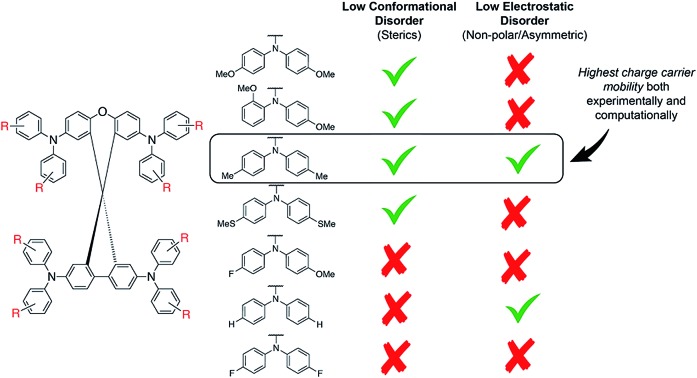
A set of design principles for high mobility xanthene-based organic hole transport materials are elucidated by combining multiple scales of theoretical chemistry (from virtual screening to bulk simulation) with experimental synthesis and characterization.

## 1 Introduction

Organic semiconducting materials are used, not only in organic electronics applications such as organic light emitting diodes (OLEDs),^1^–^3^ organic solar cells,^4^–^6^ and organic field effect transistors,^7^,^8^ but also in hybrid inorganic–organic devices such as perovskite solar cells (PSCs) where they are mainly used as printable and transparent charge extraction/blocking layers.^9^–^11^ As such, they must be affordable to synthesize, tunable in terms of their energy levels for hole and electron extraction and highly conductive and have long-term stability to optimize device performance.^12^,^13^ Systematic design of materials that fulfill these partially competing requirements requires a good understanding of the structure–property relationships and design rules of amorphous organic materials.

In this work we focus on organic-semiconductor-based hole transport materials (HTMs), a class that has been employed in perovskite solar cells.^14^–^16^ High charge carrier mobility (>10^–5^ cm^2^ V^–1^ s^–1^) and high conductivity (>10^–4^ S cm^–1^) of the HTM is essential to the performance of these materials in PSCs.^17^,^18^ Lower resistivity of the hole transport layer (HTL) in PSCs reduces the series resistance of the device and increases the fill factor.^19^,^20^ In OLEDs, higher conductivities of HTLs lowers the driving voltage of operation and increases the quantum efficiency of the device.^21^ There is a pressing need to further develop design rules to develop molecules with high mobilities and conductivities, while also retaining control over the energy level of the highest occupied molecular orbital (HOMO).

**Fig. 1:**
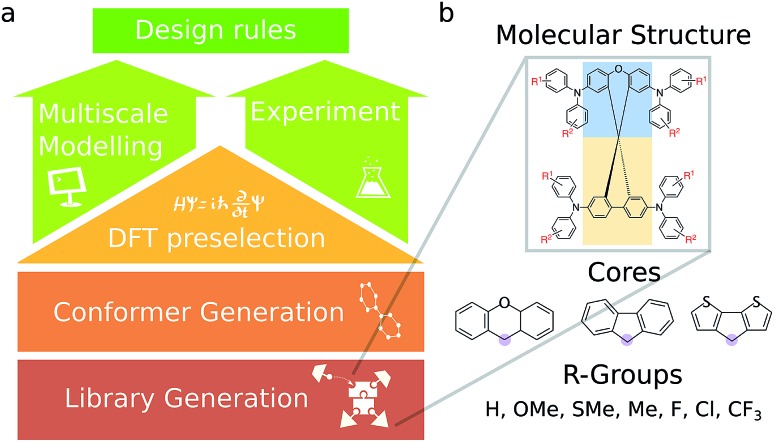
(a) The workflow employed for this study. An initial library of 590 candidates was evaluated based on frontier molecular orbitals. A sampling of molecules from this library was then selected for further experimental study and more comprehensive bulk transport property simulations. (b) Structure of molecules in the library. The molecules consist of two cores fused at a spiro center (fusion point circled in purple), functionalized at various sites, including molecules with TPA units consisting of different R-groups on the TPA units.

Here, we combine multiple scales of theoretical chemistry with experimental synthesis and characterization to elucidate a set of design principles. We virtually screen a wide library of synthesizable candidate organic semiconductors that contain modifications to the spiro-core and peripheral substituents (Fig. 1). We down-select to a series of seven analogous compounds with spiro[fluorene-9,9′-xanthene]cores on the basis of synthetic ease, low-cost starting materials, and appropriate HOMO level for use in a PSC. We systematically investigate the charge mobilities of the series of molecules by comparing to the mobility of **spiro-OMeTAD**, which is a widely used HTM in PSCs.^22^,^23^

In parallel, we conduct a state-of-the-art multiscale simulation of the hole mobilities of the selected series of **spiro-R** compounds. Recent progress in multiscale simulation of amorphous organic and metal–organic semiconductors (such as the quantum patch method)^24^–^26^ has shown that such simulations can quantitatively predict the charge carrier mobility of such materials over orders of magnitude. Here, our challenge is to examine subtle differences between HTMs from only one class of molecules whose conductivities only span two orders of magnitude, which is far more stringent than previous tests of this method. These simulations are presently too costly^27^ to be applied in a high-throughput screening approach, making a preselection of only few materials necessary.

## 2 Theoretical/computational methods

### 2.1 Virtual screening of the HTM library

An overview of the virtual screening workflow is shown in Fig. 1. The cores used for screening were assembled from xanthene, fluorene, and dithiophene moieties to form an overall spiro core, inspired by previously published HTM structures used in PSCs.^28^–^30^ To each of these cores, there were various modifications in the positions and the functional groups attached to the triphenylamine (TPA) units and the TPA substitution positions themselves. The full list of screened molecules is provided in the ESI.† Each molecule was then subject to the same computational pipeline. The conformers were generated using the RDKit package.^31^ These conformers were optimized at the B3LYP/def2-SV(P) level of theory^32^,^33^ and the frontier molecular orbital (HOMO and LUMO) energies were obtained. The Gaussian09 (ref. 34) package was used for these electronic structure calculations.

### 2.2 Bulk simulations for selected candidates

For simulation of thin film properties such as HOMO/LUMO energy distributions and hole mobility, we used the multiscale modeling approach described in Friederich *et al.*^25^,^26^ This approach includes the parameterization of molecule-specific force fields, the generation of atomistically-resolved morphologies using a Monte Carlo simulated annealing protocol,^35^ the analysis of the electronic structure of the molecules in their amorphous environment,^24^,^27^ the calculation of electronic couplings, reorganization energies, energy disorder and Marcus hopping rates and, finally, the calculation of the hole mobility using an effective medium model.^36^ We generated and analysed three morphologies (each with approximately 1000 molecules) for all seven materials to have sufficient statistics for the calculation of the hole mobility which sensitively depends on the energy disorder or the width of the distribution of HOMO energies. A detailed description of this method can be found in ref. 25.

## 3 Experimental methods

### 3.1 Synthetic procedures and characterization

**Fig. 2:**
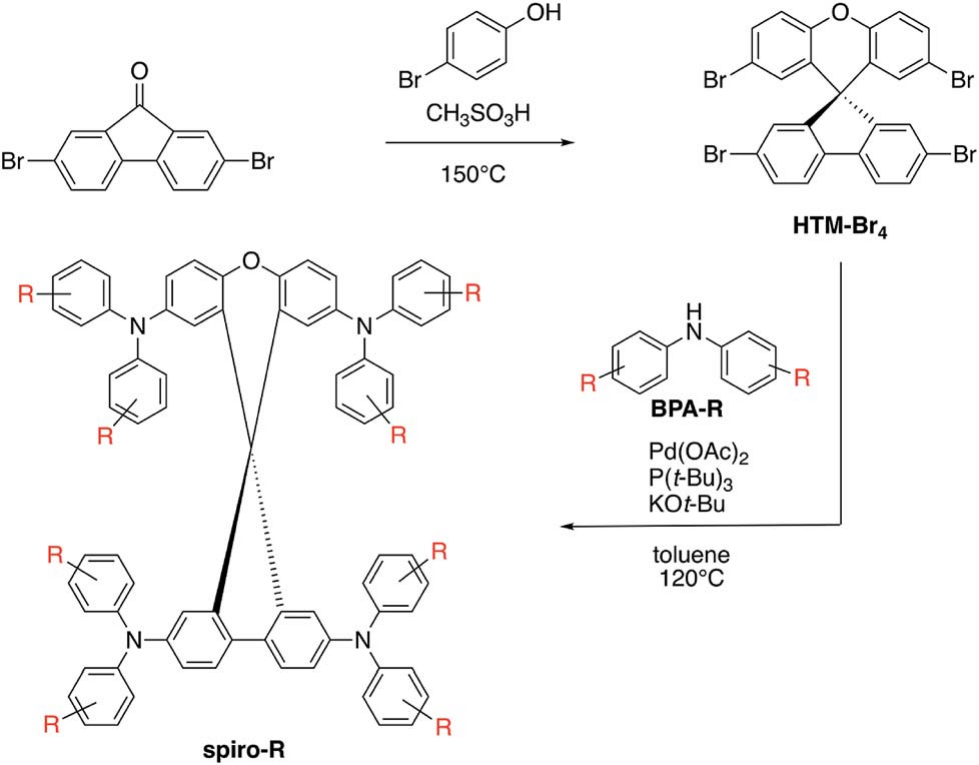
Synthetic route for **spiro-R** series, including condensation reaction for the HTM-Br_4_ core and palladium-catalyzed Buchwald–Hartwig coupling between core and secondary amine (BPA-R) for functionalized **spiro-R** products.

The series of analogous compounds were prepared following a modified literature protocol (see Fig. 2).^37^,^38^ Synthesis of the spiro core, which is consistent throughout the series, was yielded from a condensation reaction between 2,7-dibromofluorenone and 4-bromophenol to yield a spiro[fluorene-9,9-xanthene] core with four bromine substituents available for coupling (HTM-Br_4_). Secondary amines were either purchased (BPA-OMe, -Me and -H) or synthesized (BPA-*o*,*p*-OMe, -SMe, -F and -FOMe) before undergoing Pd-catalyzed Buchwald–Hartwig coupling at all four bromine positions on HTM-Br_4_ to yield the final products of the **spiro-R** series. Final compounds were purified by column chromatography and recrystallization in toluene and methanol. All intermediate and final compounds were characterized by ^1^H NMR, ^13^C NMR (Fig. S3–S12†), low and high resolution mass spectrometry.

## 4 Results and discussion

### 4.1 Hole-transport materials screening

**Fig. 3:**
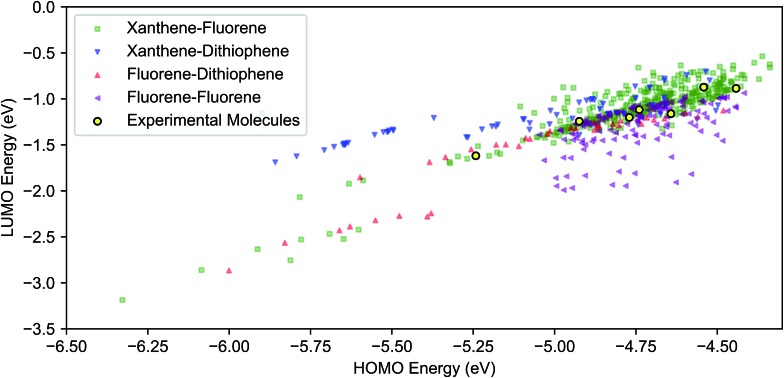
DFT (B3LYP/def2-SV(P)) HOMO and LUMO energies of 590 combinatorially-generated molecules consisting of combinations of xanthene, fluorene and dithiophene cores with different side group substitutions at different positions. The molecules that were selected for further analysis are shown in yellow. Color-coded plot with both symbols and colors corresponding to different cores.

Results of the virtual screening, focusing on the frontier molecular orbitals, are shown in Fig. 3. The calculated ranges of the HOMO and LUMO energy levels in the library are approximately 2 eV and 2.5 eV, respectively. The highest density of molecules is over approximately a 0.5 eV range for the HOMO (–5.0 to –4.5 eV) and 1 eV for the LUMO (–1.5 to 0.5 eV). With the exception of fluorene–fluorene cores, we found that the HOMO level can be tuned from –6.0 eV to –4.5 eV by varying the R-group substituents across each core type. While Fig. 3 displays the HOMO–LUMO space with respect to variation in core, insights into the effects of different functional groups can be seen in Fig. S19 and S20.† For instance, the blue strata of xanthene–dithiophene molecules to the higher LUMO/lower HOMO end of the plot correspond to molecules that lack TPA units.

### 4.2 Down-selecting from the molecular library

Selection of molecules for further theoretical modelling and experimental characterization was made on two criteria. The first criterion was the synthetic ease, corresponding to a symmetrical substitution pattern of R groups. This was successfully demonstrated on the HTM-FX molecule presented in Chiykowski *et al.*^16^ The tetrabrominated spiro[fluorene-9,9′-xanthene] core (see Fig. 2) was selected because it could be synthesized in one high yielding (>95%) condensation reaction and isolated easily by precipitation in methanol and filtration. In contrast, the core for state-of-the-art **spiro-OMeTAD** requires an air-sensitive Suzuki coupling, a moisture sensitive lithiation or Grignard reaction and a robust bromination step with column chromatographic purification.

The second criterion was the HOMO energy in vacuum, computed using density functional theory (DFT) (see Fig. 3). The selected seven molecules (**spiro-R**) represent a relatively broad range in HOMO energy levels (almost 0.75 eV), and more importantly, contain variations in the side groups that can change both the electronic landscape and the morphology. The selected molecules span the range of the highest density region of our screened frontier molecular orbital space shown in Fig. 3.

### 4.3 CV, optical measurements and comparison to simulations

The selected seven compounds were characterized by UV-Vis absorption and emission spectroscopy (Fig. S1†). The spectra of all seven HTMs display an absorption onset between 400–420 nm. It should be noted that this is appropriate for use in solar cell and OLED applications as these materials do not absorb significantly in the visible region and will not compete with absorption or emission processes of the photoactive components of the device. The materials all exhibit similar absorption features indicative of their analogous electronic structure and allowed transitions.

**Fig. 4:**
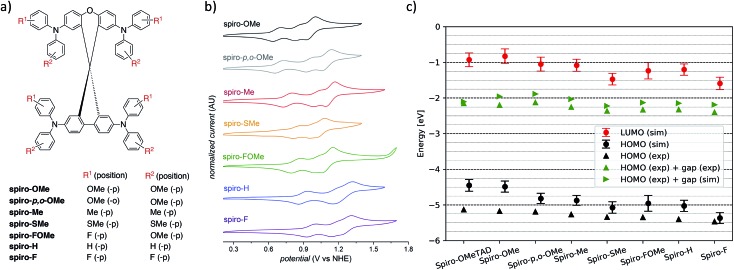
(a) Definition of the functional groups used for the **spiro-R** series. (b) Cyclic voltammograms for HTM series recorded in 0.1 M *n*-NBu_4_PF_6_ DCM solutions at room temperature. (c) Simulated and experimental HOMO and LUMO energies for the **spiro-R** series and **spiro-OMeTAD** as a reference material.

Cyclic voltammograms (CVs) for the series of seven HTMs were recorded in 0.1 M *n*-NBu_4_PF_6_ DCM solutions to determine the reduction potential (*E*_HOMO_), which we can compare to the computed HOMO energies (*ε*_HOMO_Fig. 4b and Table S1†). The first oxidation (*E*_HOMO_) of these materials occurred between 0.67 to 0.96 V *vs.* NHE, with a clear trend that increasing electron-withdrawing character of substituents increases *E*_HOMO_. The trend observed for the experimentally determined *E*_HOMO_ is fully supported by the HOMO energies determined from the vacuum calculations and bulk simulations (Fig. 4c and Table S1†) and confirms the role of terminal substituents on tuning the electrochemical properties of organic HTMs. Faradaic events occurring at potentials higher than *E*_HOMO_ likely correspond to the remaining TPA units on the molecules, though these oxidations following the first oxidation are not necessarily reversible.

### 4.4 Conductivity measurements

The intrinsic conductivities of spiro-based HTMs, like **spiro-OMeTAD**, can be low (generally <10^–4^ S cm^–1^).^39^,^40^ and typically benefit from chemical oxidation through p-doping to increase the number of charge carriers present in the material. LiTFSI is a widely used p-type dopant for HTLs layers of perovskite solar cells.^41^

Conductivity measurements were performed on films of each of the seven compounds doped with 20 mol% LiTFSI and containing 300 mol% *tert*-butylpyridine (*t*BP) to ensure good film morphologies. Results in Table 1 show that **spiro-OMeTAD** (with 20 mol% LiTFSI) has a conductivity of 1.23 × 10^–4^ S cm^–1^, consistent with literature reports.^42^,^43^ **Spiro-OMe**, **spiro-*p*-,*o*-OMe** and **spiro-SMe**, which contain isoelectronic substituent groups, are a factor of 2–3 lower. **Spiro-Me** exhibits the highest conductivity (2.47 × 10^–4^ S cm^–1^) of the series. Asymmetrically substituted **spiro-FOMe** exhibited the lowest conductivity, followed by small, highly polar **spiro-F**. Conductivity data was also collected with 20 mol% LiTFSI, 3 mol% CoTFSI and 300 mol% tBP but no trend in the effect of CoTFSI doping on conductivity was found (Table S2†).

**Table 1:** Experimental conductivity data for **spiro-OMeTAD** and the **spiro-R** series

Name	Conductivity [S cm^–1^] (doped with 20% LiTFSI)
**spiro-OMeTAD**	1.23 × 10^–4^
**spiro-OMe**	5.81 × 10^–5^
**spiro-*p*-,*o*-OMe**	3.72 × 10^–5^
**spiro-Me**	2.47 × 10^–4^
**spiro-SMe**	6.92 × 10^–5^
**spiro-FOMe**	1.90 × 10^–6^
**spiro-H**	2.82 × 10^–5^
**spiro-F**	1.57 × 10^–5^

### 4.5 Theoretical modelling of mobility

The relationship between conductivity *σ* and mobility *μ* is

1 *σ* = *neμ*(*n*),

where *n* is the charge carrier density, *e* is the charge of an electron and *μ*(*n*) is the (hole) mobility which depends on the charge carrier density. We assume a charge carrier density of one hole per 100 molecules^44^ and an use our simulated morphologies for the molecular density. Simulated densities and density profiles for the morphologies can be found in Table S4 and Fig. S17.† There are two effects which influence the mobility as a function of *n*. The first is a trap filling effect.^45^ Depending on the energy disorder, the mobility increases by approximately one order of magnitude upon going from low hole densities (10^–5^) to 10^–2^ holes per molecule. This effect only considers additional holes but neglects the influence of ionized dopants on the mobility. Thus, a second effect influences the dependence of the hole mobility on the hole density: the ionized dopants increase the (electrostatic) energy disorder and thus reduce the mobility. The two opposite effects partially cancel each other (depending on the value of the energy disorder) but the quantification of both effects remains to be shown.

**Fig. 5:**
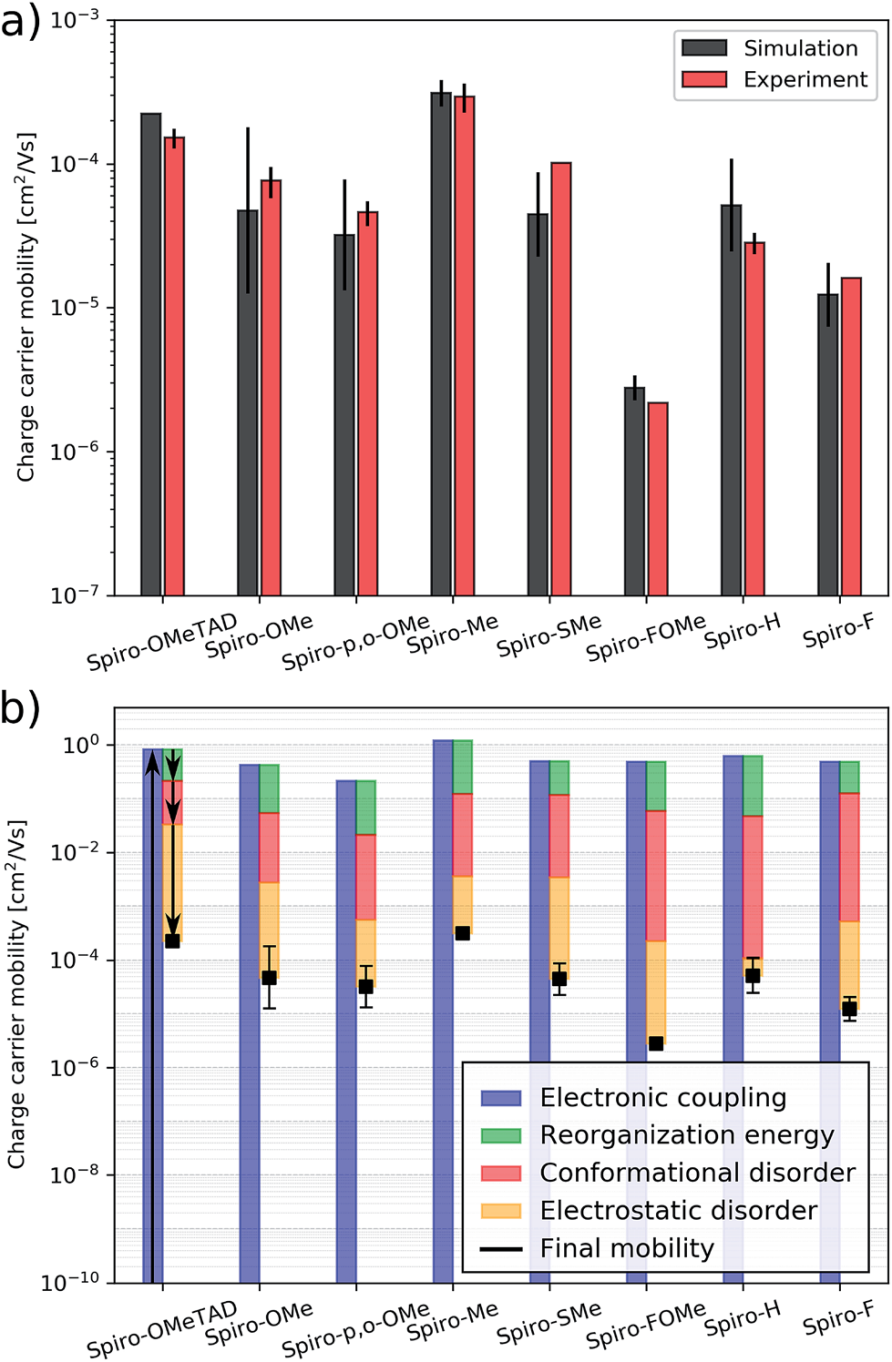
(a) Simulated and measured hole mobility of **spiro-OMeTAD** and the **spiro-R** series, (b) hole mobility of **spiro-OMeTAD** and the **spiro-R** and its partitioning in effects of electronic couplings, reorganization energy, conformational disorder and electrostatic disorder.

The HTMs studied here have similar energy disorder parameters. We assume that doping is similarly efficient in all of the materials. The effects described above will therefore be of similar strength throughout the entire HTM series, which allows us to neglect the influence of the hole density and ionized dopants on the mobility. Assuming a linear relation between the hole mobility, and hole density and conductivity (eqn (1)) enables us to compare the experimental conductivities shown in Table 1 with the simulated hole mobilities (see Fig. 5a). We find a good agreement between the computed and experimental values for the entire HTM series including the reference material **spiro-OMeTAD**. The hole mobility of **spiro-OMeTAD** was measured in previous experimental studies to be approximately 2 × 10^–4^ cm^2^ (V^–1^ s^–1^),^46^ which is in good agreement with the simulated value of 2.2 × 10^–4^ cm^2^ (V^–1^ s^–1^).

The simulation protocol allows for us to decompose the contributions to the overall mobility (Fig. 5b); we are able to distinguish and quantify the influence of various microscopic characteristics on the hole mobility. These include electronic couplings and reorganization energies as well as two sources of energy disorder: (1) conformational disorder due to geometrical differences between the molecules in the amorphous structure and (2) electrostatic disorder due to the electrostatic interaction between molecules with their amorphous environment. As shown in previous work,^25^ the electronic coupling strength can be used to calculate an upper bound of the charge carrier mobility which is then reduced by reorganization and energy disorder. We find that the molecules with nonpolar side groups (*e.g.***spiro-Me** and **spiro-H**) have small values of electrostatic disorder; this has a positive influence on the charge carrier mobility. This design rule was already discussed and exploited in ref. 26 to systematically increase the electron mobility of organic semiconductors. Polar substitution, in particular fluorination (*e.g.***spiro-FOMe** and **spiro-F**), has the opposite effect: the electrostatic disorder increases and the hole mobility decreases. Therefore, **spiro-Me** outperforms **spiro-H** due to the large conformational disorder in **spiro-H** according to the simulation results. A systematic analysis of all seven **spiro-R** materials indicates that the size of the –R group influences the conformational disorder. Larger side groups, in particular methoxy groups (independent of their position) limit internal conformation changes and thus reduce the conformational disorder while smaller side groups such as hydrogen and fluorine correlate with higher values of the conformational disorder. This correlation can be used as a design rule for future HTM optimization to systematically reduce conformational disorder and thus increase mobility. The reorganization energy, which is often prominently considered in organic electronics applications, in particular in crystalline materials, is largely similar across this set of molecules and for this backbone does not appear to be a determining factor in design.

## 5 Conclusion

Spiro[fluorene-9,9′-xanthene]-based HTMs are a synthetically accessible class of organic semiconductors for organic electronics. A computational survey of the frontier molecular orbital landscape shows that these materials are capable of accessing a large range of HOMO and LUMO energies, comparable to that of molecules with both the traditional spirobifluorene core and spirodithiophene cores. This diversity in *E*_HOMO_ has been confirmed by the synthesis and characterization of a series of seven **spiro-R** HTM derivatives.

We find that the major consideration when designing HTMs, after accounting for the HOMO and LUMO levels, is to reduce the contributions from conformational and electrostatic disorder to achieve high hole mobilities. We found that larger functional groups, such as methyl and methoxy groups, systematically reduce the conformational disorder. Functional groups with higher polarity or asymmetry in substitution will also increase the electrostatic disorder of the material, reducing the hole mobility (*e.g.*, thiomethyl and -*p*,*o*-methoxy groups).

Future models for doping must take into account both the affinity of the material to the dopant and the propensity of the dopant to increase the charge carrier density. For this class of similar materials, we found that our assumption that differences in dopant affinity with Li^+^ were not appreciable and we were able to draw a direct link between bulk simulations of mobilities and measured conductivities. Bulk simulations are still computationally expensive for these materials, so the ability to draw broader conclusions will obviate the need to do simulations for all possible materials in the future. This also informs what molecules to focus on for future large-scale molecular screening studies of HTMs.

## Conflicts of interest

There are no conflicts of interest to declare.

## Supplementary Material

Supplementary information

Supplementary information
