# Supplementary material for: Design rules for high mobility xanthene-based hole transport materials
Source: Chem Sci. 2019 Jul 25;10(36):8360–6. doi: 10.1039/c9sc01491h (PMC6839588; doi:10.1039/c9sc01491h)
Supplement: Supplementary file 1 [file SC-010-C9SC01491H-s001.pdf]

Electronic Supplementary Material (ESI) for Chemical Science.
This journal is © The Royal Society of Chemistry 2019

Electronic Supplementary Information for "Design Rules for High Mobility Xanthene-Based Hole Transport Materials"

Daniel P. Tabor,[†] Valerie A. Chiykowski,[‡] Pascal Friederich,[†,¶,§] Yang Cao,[‡,‖] David J. Dvorak,[‖] Curtis P. Berlinguette,[*,‡,‖,⊥] and Alán Aspuru-Guzik[*,†,¶,#,@]

†*Department of Chemistry and Chemical Biology, Harvard University, 12 Oxford St., Cambridge, MA, 02138, USA*

‡*Department of Chemistry, University of British Columbia, 2036 Main Mall, Vancouver, BC, V6Y 1Z1, Canada*

¶*Department of Chemistry, University of Toronto, 80 St. George Street, Toronto, ON M5S 3H6, Canada*

§*Institute of Nanotechnology, Karlsruhe Institute of Technology, Hermann-von-Helmholtz-Platz 1, 76344 Eggenstein-Leopoldshafen, Germany*

‖*Stewart Blusson Quantum Matter Institute, The University of British Columbia, 2355 East Mall, Vancouver, BC, V6T 1Z4, Canada*

⊥*Department of Chemical and Biological Engineering, The University of British Columbia, 2360 East Mall, Vancouver, BC, V6Y 1Z3, Canada*

#*Department of Computer Science, University of Toronto, 214 College St, Toronto, ON M5T 3A1, Canada*

@*Vector Institute, 661 University Ave Suite 710, Toronto, ON M5G 1M1, Canada*

E-mail: cberling@chem.ubc.ca; alan@aspuru.com

Contents

S1 Spectral and Electrochemical Characterization of Spiro-R Series S3

S2 Bulk Thermal Properties of Spiro-R Series S6

S3 NMR Characterization of Spiro-R Series S7

S4 Synthetic Procedures S15

S5 Packing Density Analysis from Bulk Simulation S21

S6 Correlation Plots Between Experimental and Simulated Values S22

S7 Calculated Frontier Molecular Orbital Properties Based on Functional Groups S22

S8 Frontier Molecular Orbital Energies in Vacuum and in Implicit Solvent S24

S1 Spectral and Electrochemical Characterization of Spiro-R Series

Optical and Electrochemical Properties

Solution UV-visible absorption spectra were recorded using a Cary 5000 UV/vis spectrophotometer. Photoluminescence (PL) spectra were recorded with a Cary Eclipse. All samples were measured in a 1 cm cell at room temperature with DCM as solvent. Concentration of 2×10^{-5} M and 1×10^{-5} M were used for solution UV/visible and PL, respectively. Solution-phase electrochemical data was recorded with a CHI660D potentiostat using a platinum wire counter electrode, Ag/AgCl reference electrode, and a platinum working electrode. A 0.1 M n-NBu4PF6 electrolyte solution in DCM at ambient temperature was used for all HTMs. Ag/AgCl in saturated KCl was used as the reference electrode and was calibrated versus the normal hydrogen electrode (NHE) by addition of 0.235 mV. Cyclic voltammograms (CVs) were acquired for 0.5 mM solutions of each HTM at a scan rate of 50 mV s^{-1}.

Conductivity Measurements

Four parallel Au electrodes with a spacing of 0.75 mm and length of 23 mm were used to measure the film conductivities in the dark and under ambient conditions. A Keithley 2400 sourcemeter was used to force current through the outer electrodes while sensing the voltage across the inner two electrodes. A linear fit of each measurement was used to determine the resistance, R, and the sample geometry was then used to calculate the conductivity, σ, according to the equation:

$$\sigma = \frac{d}{Rlt} \tag{1}$$

where l is the electrode length, d is the inter-electrode spacing and t is the film thickness. The film thickness was measured using a Bruker DektakXT profilometer.

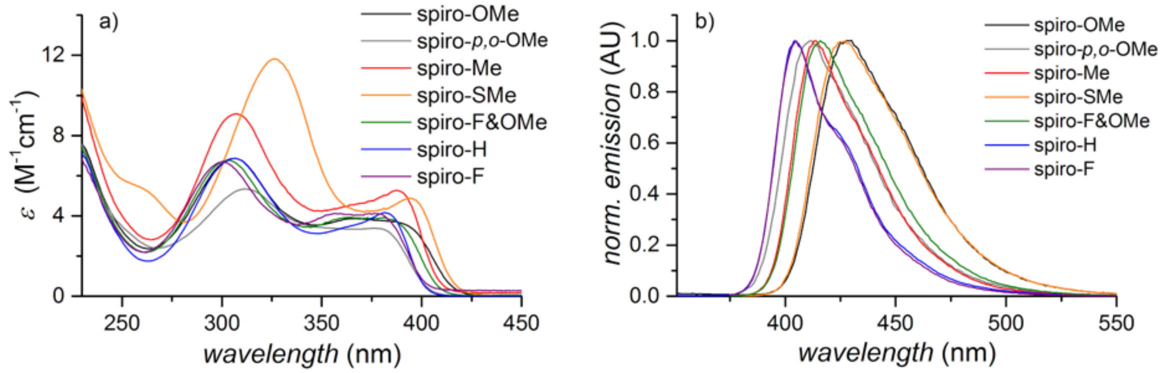

Figure S1: (a) UV-Visible absorption spectra and (b) emission spectra for **spiro-R** series recorded in DCM.

Table S1: Summary of experimental spectroscopic and electrochemical properties for the **spiro-R** series

Compound	λ^a_{max} (nm)	λ^b_{em} (nm)	E^c_{gap} (eV)	E^d_{HOMO} (V vs NHE)	ϵ^f_{HOMO} (eV)	ϵ^g_{LUMO} (eV)
**spiro-OMe**	306, 365	429	2.97	0.67	-5.17	-2.30
**spiro-*p*-,*o*-OMe**	313, 354	413	3.07	0.67	-5.19	-2.38
**spiro-Me**	307, 388	414	3.02	0.77	-5.27	-2.25
**spiro-SMe**	327, 395	427	2.97	0.84	-5.34	-2.13
**spiro-FOMe**	303, 381	416	3.02	0.85	-5.35	-2.17
**spiro-H**	306, 381	405	3.08	0.90	-5.40	-2.18
**spiro-F**	300, 376	405	3.06	0.96	-5.46	-2.10

[a] Absorption maximum for HTM in DCM. [b]Excitation at λ_{max} of higher energy/lower wavelength. [c]Determined from the intersection of absorption and emission spectra. [d]The half-wave potential from first oxidation of cyclic voltammogram of HTM in DCM referenced to NHE. [f]$\epsilon_{HOMO} = -(E_{HOMO} + 4.50\text{ eV})$. [g]The LUMO determined from the ϵ_{HOMO} and E_{gap}

Table S2: Conductivity data for **spiro-OMeTAD** and the **spiro-R** series

Compound	Conductivity 20% LiTFSI$^+$ (S/cm)	Conductivity 20% Li$^+$ and (S/cm)
**spiro-OMeTAD**	1.23×10^{-4}	1.67×10^{-4}
**spiro-OMe**	5.81×10^{-5}	2.11×10^{-4}
**spiro-*p*-,*o*-OMe**	3.72×10^{-5}	2.20×10^{-5}
**spiro-Me**	2.47×10^{-4}	1.17×10^{-5}
**spiro-SMe**	6.92×10^{-5}	3.75×10^{-6}
**spiro-FOMe**	1.90×10^{-6}	4.32×10^{-5}
**spiro-H**	2.82×10^{-5}	7.43×10^{-6}
**spiro-F**	1.57×10^{-5}	1.67×10^{-4}

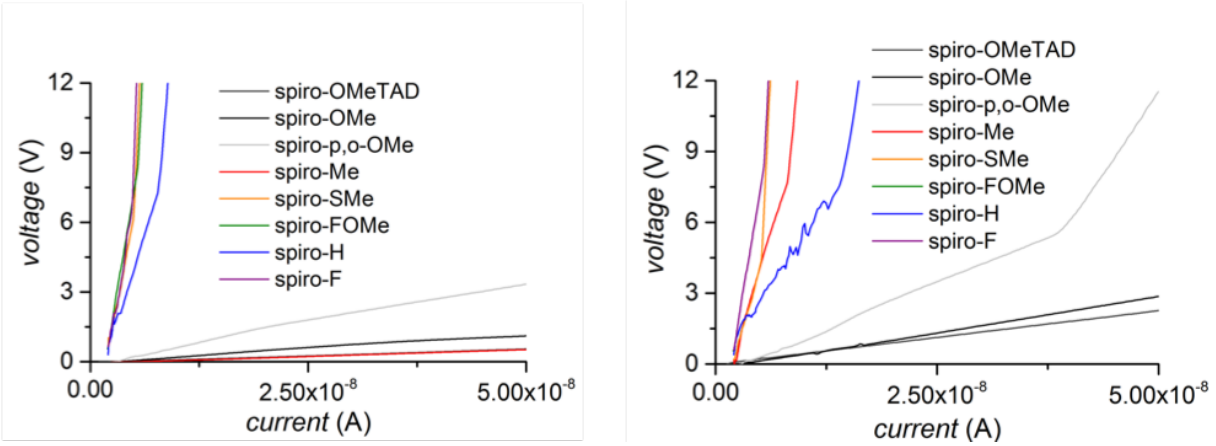

Figure S2: Resistivity data for **spiro-OMeTAD** and **spiro-R** series with a) 20% Li and b) 20% Li and 3% Co doping

S2 Bulk Thermal Properties of Spiro-R Series

Thermal Properties DSC (Differential Scanning Calorimetry) curves of compounds were collected using a Netzsch DSC 214 Polyma instrument under a nitrogen protective atmosphere. For each measurement, two aluminum crucibles were used, one as empty reference and the other for sample measurement. A customized heating program was developed to include a 5 min hold at the initial temperature of 50 °C and four consecutive heating-cooling cycles (Tmin = 50 °C, Tmax = 300 °C, 10 K/min). Data from the last 3 heating segments were used to identify the T_m (melting point) and extrapolate the T_g (glass transition temperature) of the material.

Table S3: Summary of bulk thermal properties for the **spiro-R** series

Compound	T_g^a (°C)	T_m^a (°C)
**spiro-OMe**	106.7	> 300
**spiro-*p*-,*o*-OMe**	101.3	> 300
**spiro-Me**	127.3	> 300
**spiro-SMe**	125.5	> 300
**spiro-FOMe**	96.4	> 300
**spiro-H**	98.2	> 300
**spiro-F**	113.6	> 300

[a] Determined by differential scanning calorimetry (DSC).

S3 NMR Characterization of Spiro-R Series

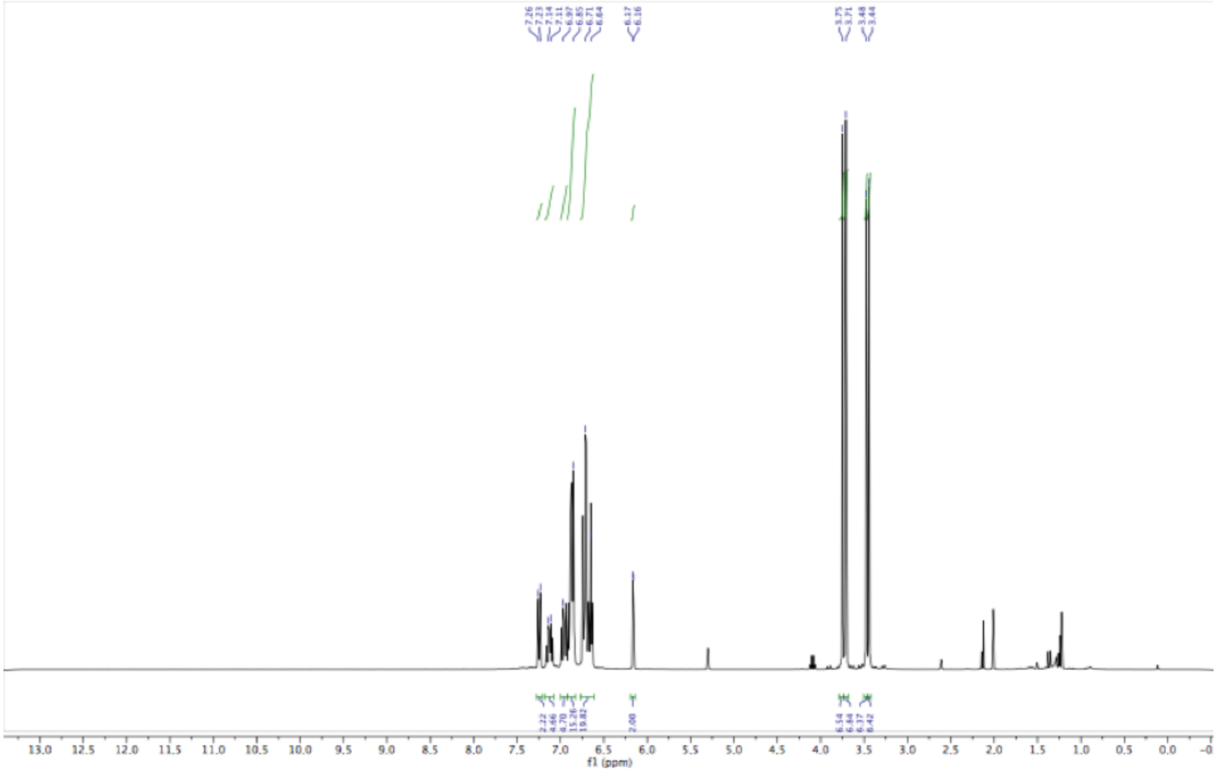

Figure S3: ^{1}H NMR of **spiro-*p*-,*o*-OMe** in CD_2Cl_2 at ambient temperature.

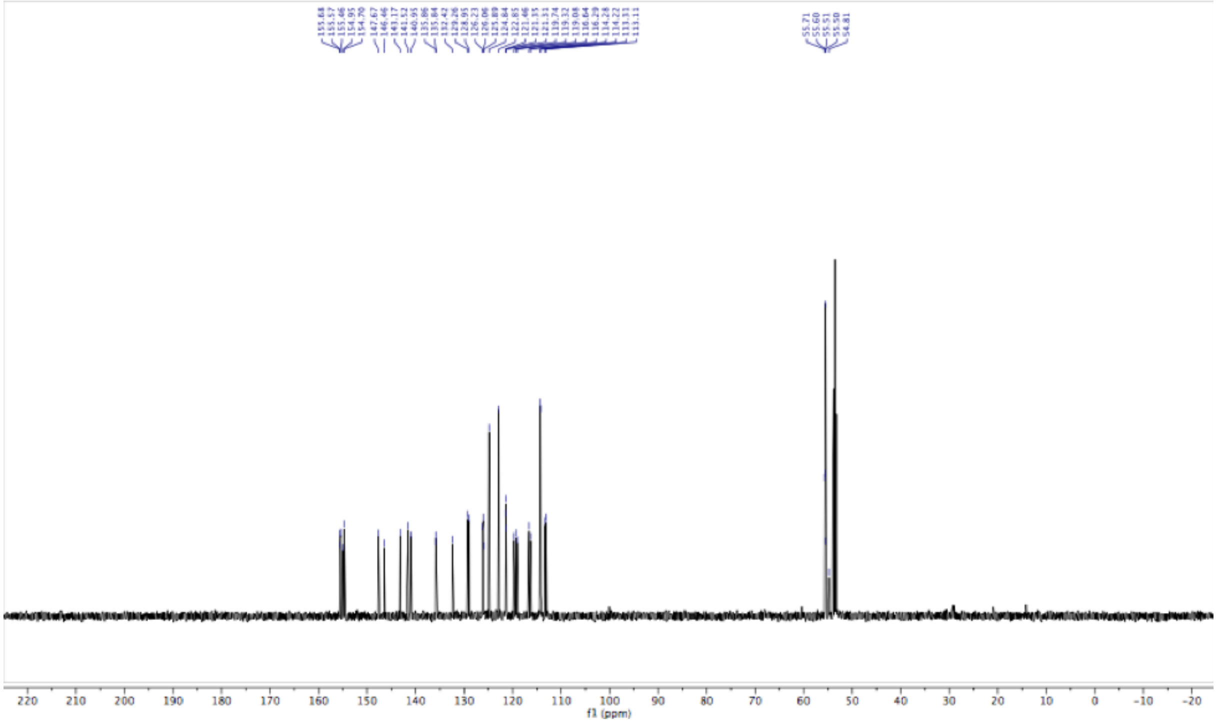

Figure S4: ^{13}C NMR of **spiro-*p*-,*o*-OMe** in CD_2Cl_2 at ambient temperature.

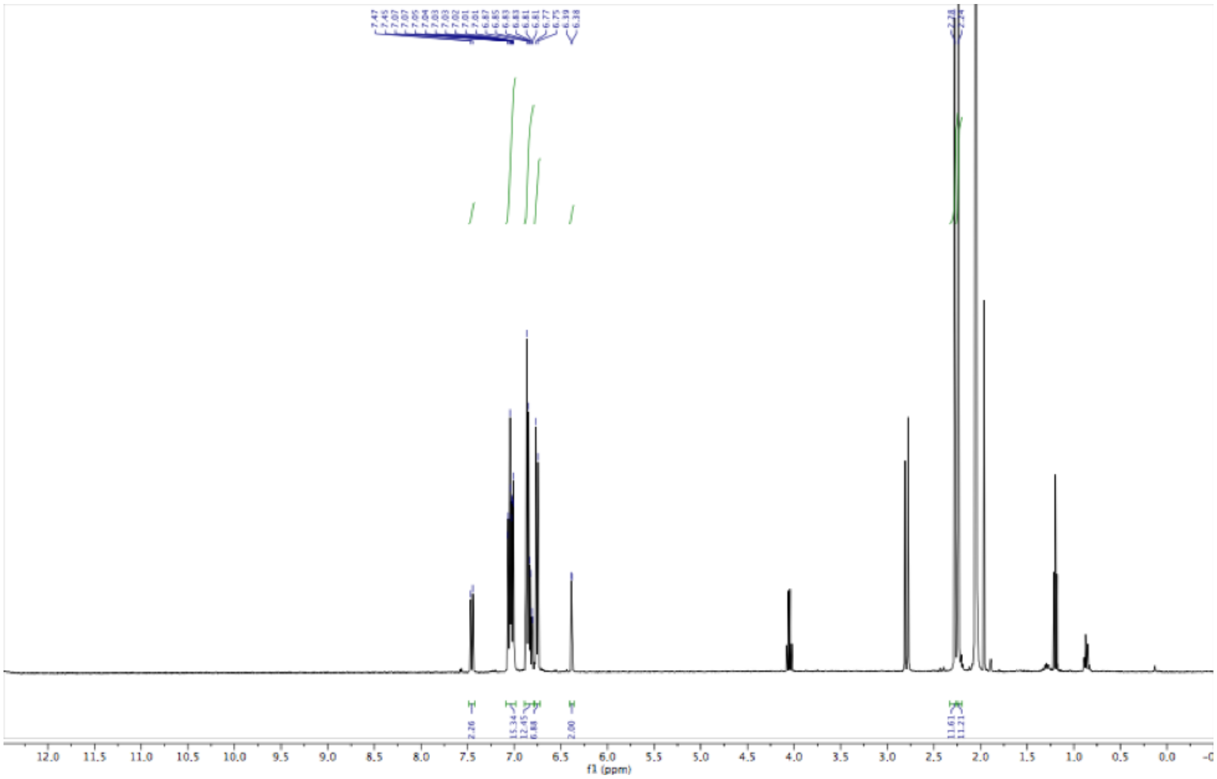

Figure S5: ^{1}H NMR of **spiro-Me** in CD_2Cl_2 at ambient temperature.

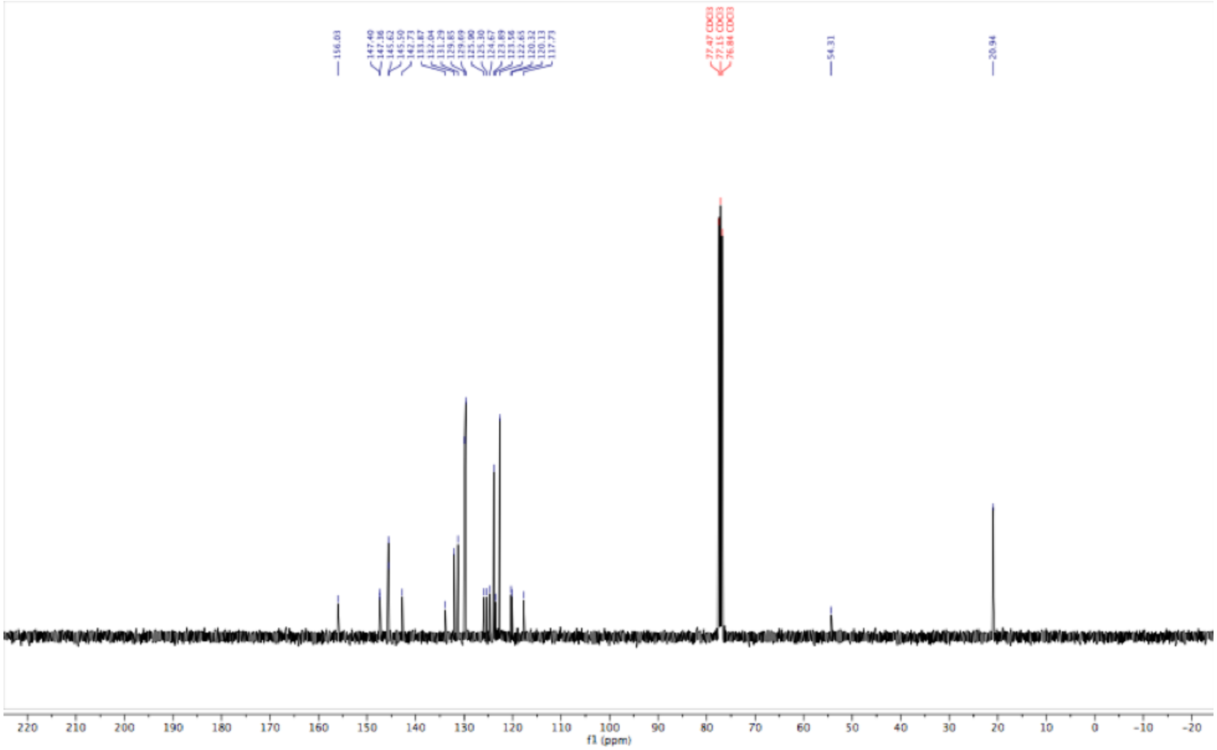

Figure S6: ^{13}C NMR of **spiro-Me** in CD_2Cl_2 at ambient temperature.

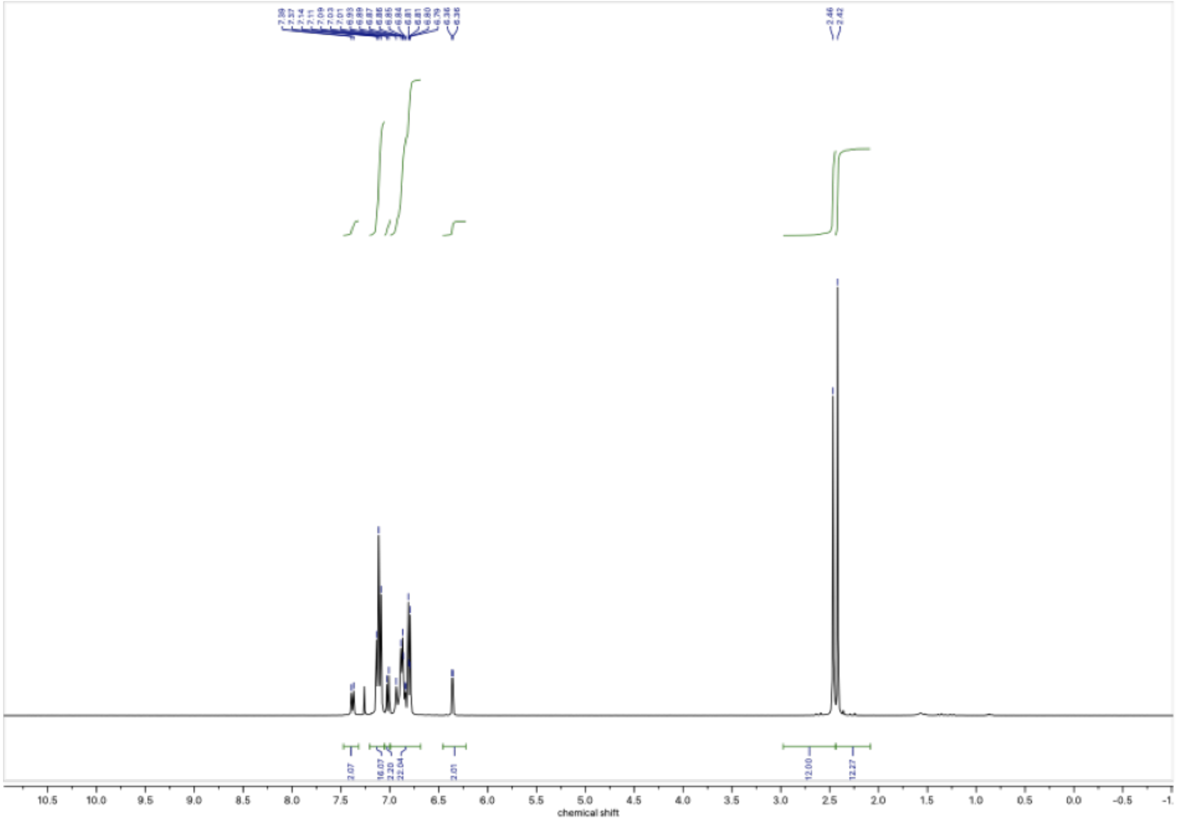

Figure S7: ^{1}H NMR of **spiro-SMe** in $CDCl_3$ at ambient temperature.

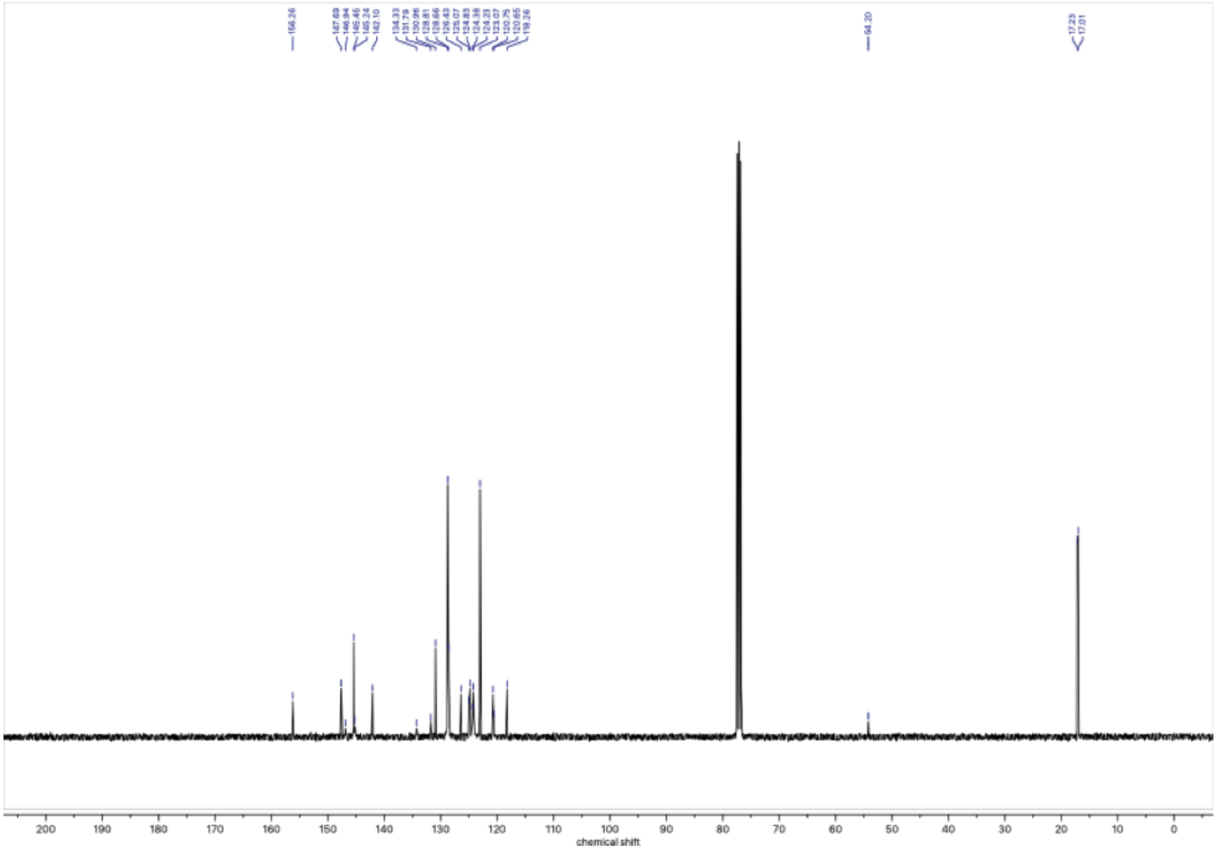

Figure S8: ^{13}C NMR of **spiro-SMe** in $CDCl_3$ at ambient temperature.

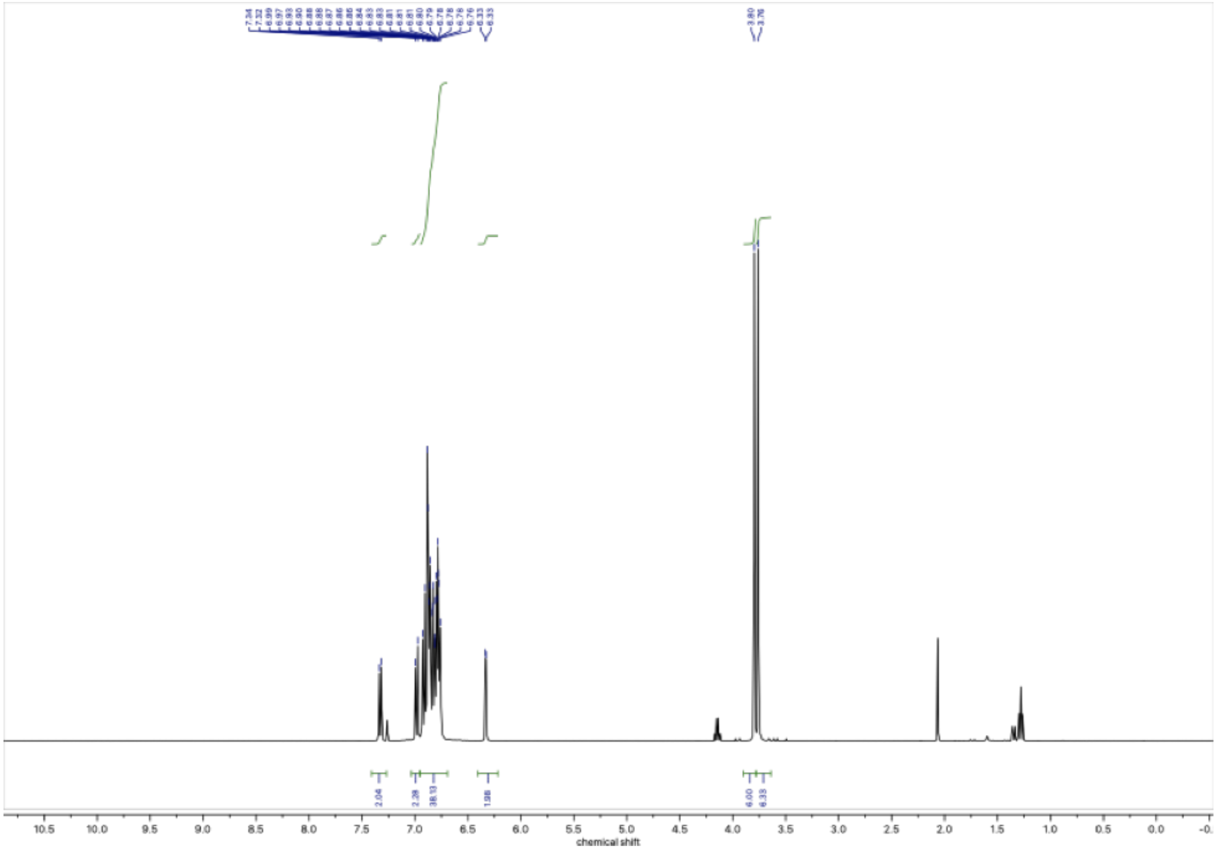

Figure S9: ^{1}H NMR of **spiro-FOMe** in $CDCl_3$ at ambient temperature.

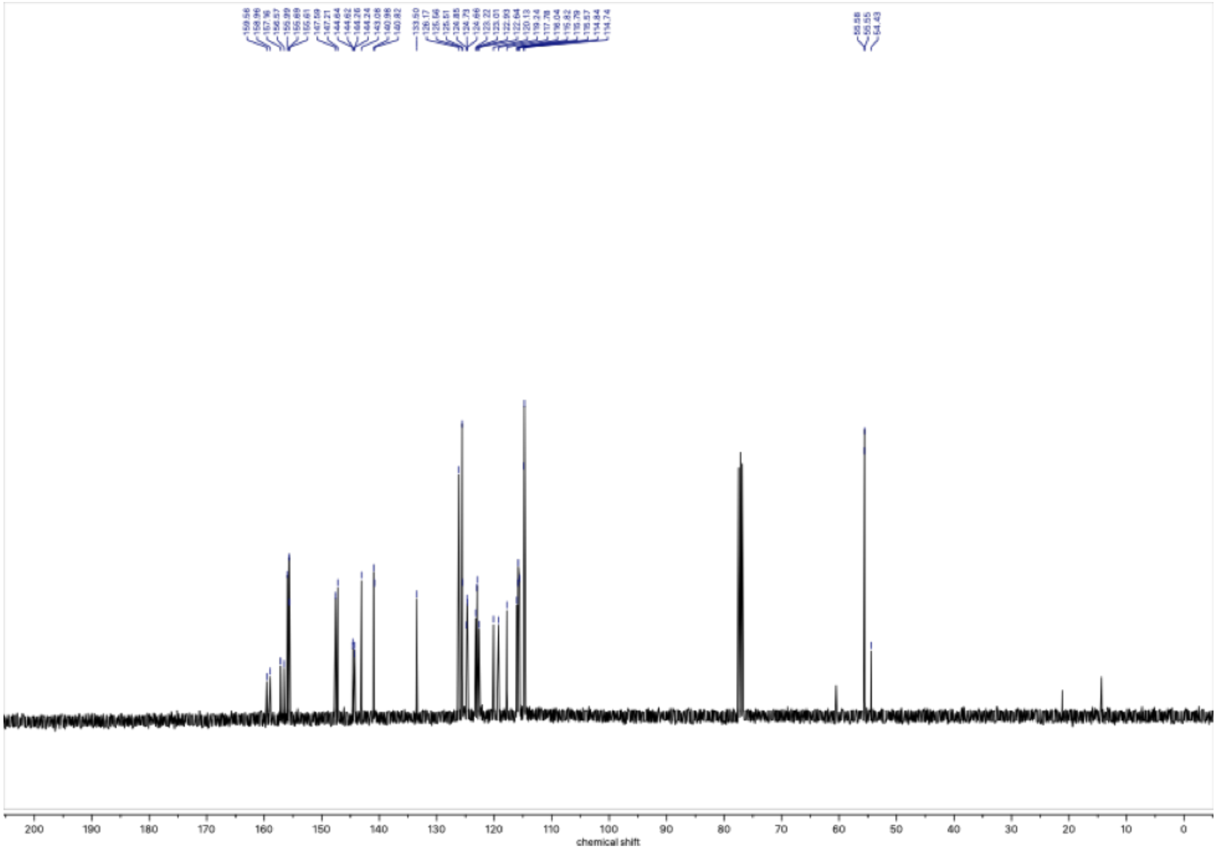

Figure S10: ^{13}C NMR of **spiro-FOMe** in $CDCl_3$ at ambient temperature.

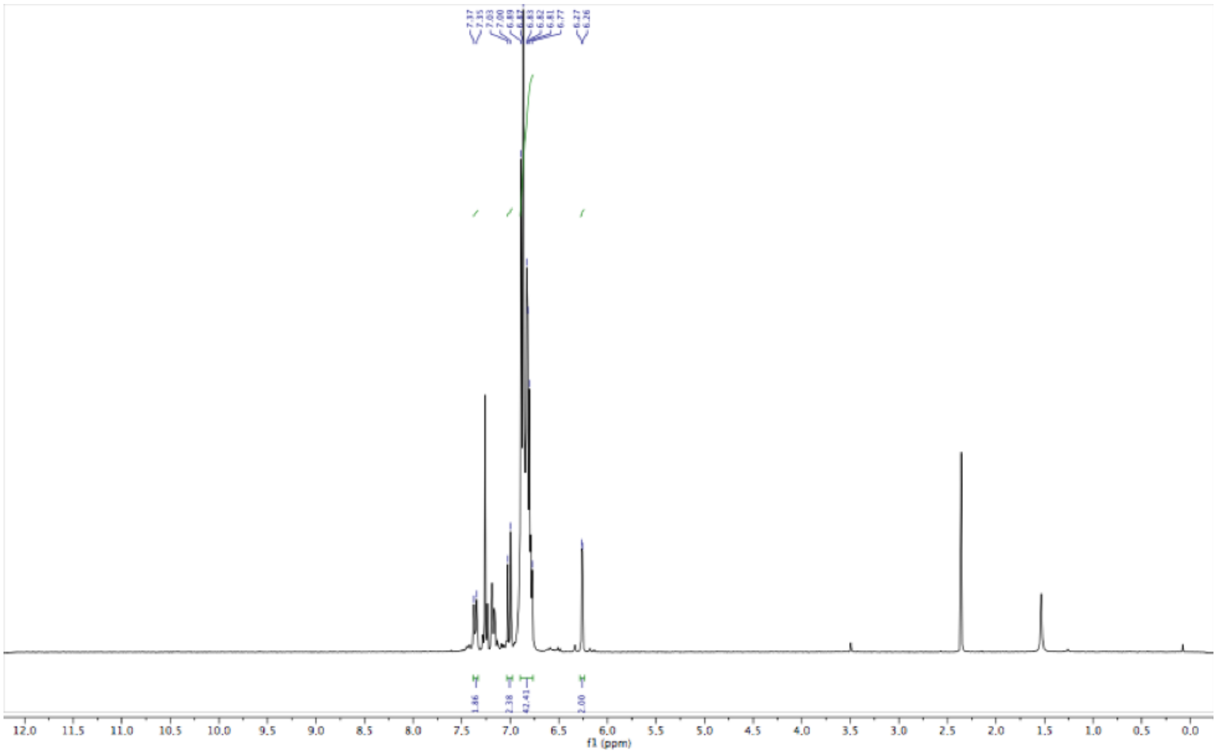

Figure S11: ^{1}H NMR of **spiro-F** in CD_2Cl_2 at ambient temperature.

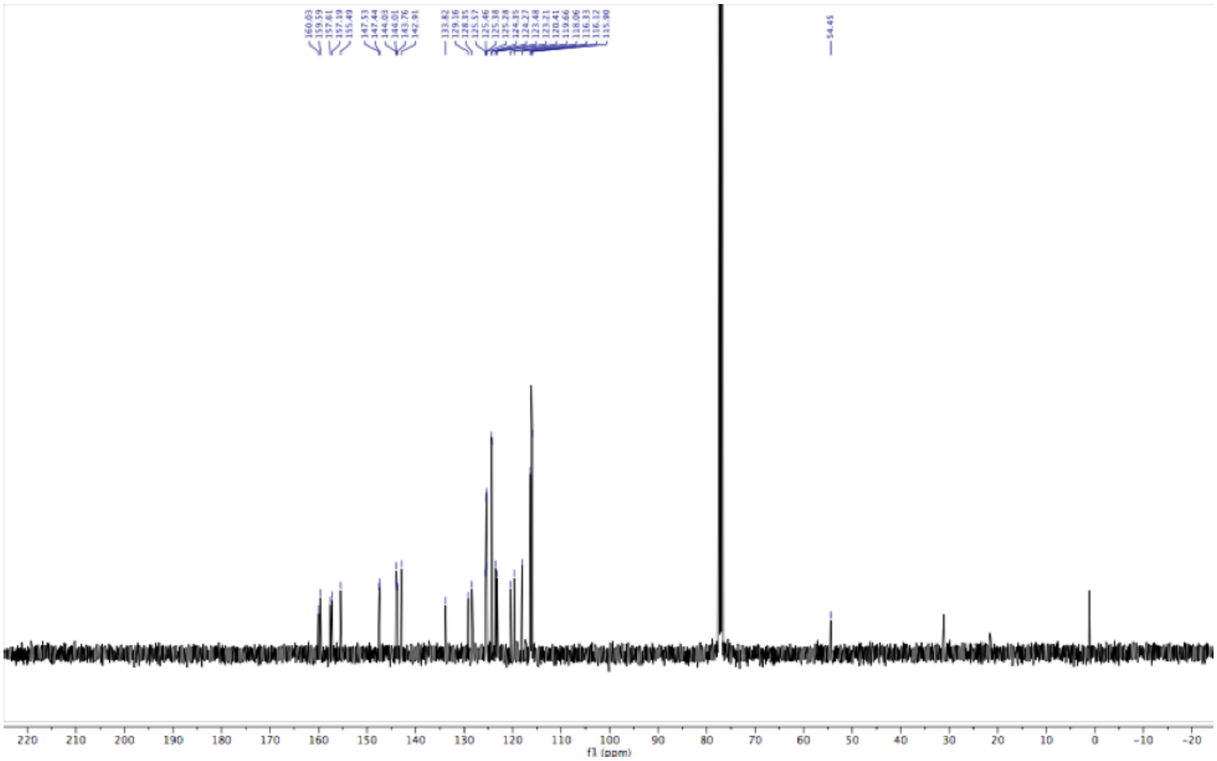

Figure S12: ^{13}C NMR of **spiro-F** in CD_2Cl_2 at ambient temperature.

S4 Synthetic Procedures

Preparation of compounds. All reagents were obtained from Sigma Aldrich, Alfa Aesar or Fisher Scientific and used as received. All reactions were performed using toluene that was passed through a solvent purification system prior to use. Standard inert atmosphere and Schlenk techniques were carried out under nitrogen. All reactions and purification were carried out in the dark. Purification by column chromatography was performed using silica (Silicycle: Ultrapure Flash Silica). Analytical thin-layer chromatography (TLC) was performed on aluminum-backed sheets pre-coated with silica 60 F-254 adsorbent (250 μm thick; Silicycle, QC, Canada) and visualized under UV light. Routine ^{1}H and ^{13}C NMR spectra were collected on a Bruker AV300 or Bruker AV400 inv/dir instrument at ambient temperatures, operating at 400 MHz and 100 MHz, respectively. Chemical shifts (δ) are reported in parts per million (ppm) using the residual signals δ 7.26 and 77.0 for $CDCl_3$, δ 2.05 and 29.8 for Acetone-d6 and δ 2.50 and 39.4 for DMSO-d6 as internal references for ^{1}H and ^{13}C, respectively.

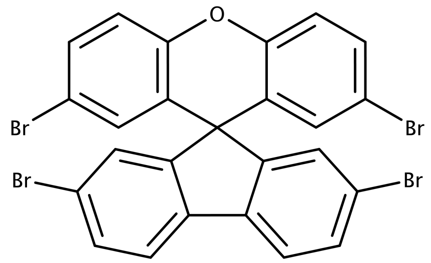

Figure S13: HTM-Br4

HTM-Br4 (2,2',7,7'-tetrabromospiro[fluorene-9,9'-xanthene]): 2,7-dibromo-9-flourenone (1.08 g, 3.20 mmol), 4-bromophenol (5.53 g, 32.0 mmol) and methane sulfonic acid (0.82 mL, 12.8 mmol) were mixed at 140C for 18 h under N2. The reaction mixture was cooled to room temperature and product was precipitated with the addition of MeOH. Separation by filtration and rinsing with MeOH yielded 2.09 g (96.5%) of product as white powder ^{1}H NMR (300 mHz, Acetone-d6) δ = 8.01 (d, J = 8.1 Hz, 1 H), 7.69 (dd, J = 8.2, 1.8 Hz, 1H), 7.49 (dd, J = 8.8, 2.4 Hz, 1H), 7.44 (d, J = 1.7 Hz, 1 H), 7.31 (d, J = 8.8 Hz, 1H), 6.53 (d, J = 2.4 Hz, 1H). ^{13}C NMR (100 MHz, Acetone-d6): δ= 155.52, 149.95, 137.57, 132.20, 130.39, 129.05, 124.79, 122.89, 121.96, 119.16, 116.20 ppm. HRMS

(EI) m/z: 643.76141 [(M+)] calcd for $C_{25}H_12O_{79}Br_4$ m/z: 643.76216.

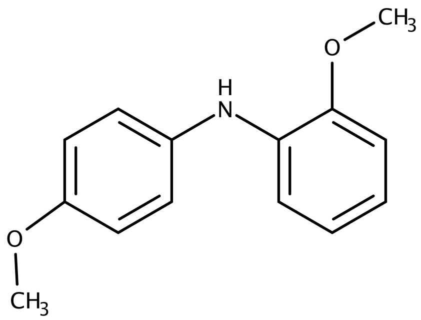

Figure S14: BPA-p,o-OMe

BPA-p,o-OMe (2-Methoxy-N-(4-methoxyphenyl)aniline): 2-iodoanisole (1.00 g, 4.27 mmol), p-anisidine (0.53 g, 4.27 mmol), palladium acetate (0.061 g, 0.30 mmol), tri-tert-butylphosphine (0.110 g, 0.491 mmol) and potassium tert-butoxide (1.03 g, 10.7 mmol) were added to toluene (15 ml) under N2. The reaction mixture was stirred at reflux for 16 h. The reaction mixture was cooled to room temperature. Purification by silica column chromatography (SiO_2: hexanes/EtOAc, 8:1) yielded 0.807 g (81%) of pale yellow solid product. ^{1}H NMR (400 mHz, $CDCl_3$) $\delta = 7.12$, ((d, J = 8.8 Hz, 1 H), 7.04 (dd, J = 7.7, 1.7 Hz, 1H), 7.05-6.77 (m, 6H), 5.97 (s, 1H). ^{13}C NMR (100 MHz, Acetone-d6): $\delta = 155.75$, 147.83, 135.88, 135.52, 123.22, 121.39, 119.00, 115.06, 113.07, 110.68, 56.05 ppm. HRMS (EI) m/z: 230.1190 [(M+)] calcd for $C_{14}H_{16}NO_2$ m/z: 230.1181.

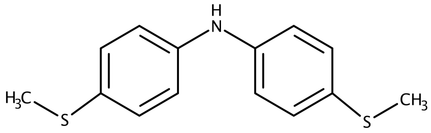

Figure S15: BPA-SMe

BPA-SMe (Bis(4-(thiomethyl)phenyl)amine: (4-bromophenyl)(methylsulfane (2.03 g, 10.0 mmol), 4-(methylthioaniline) (1.39 g, 10.0 mmol), palladium acetate (0.112 g, 0.50 mmol), tri-tert-butylphosphine (0.100 g, 0.5 mmol) and potassium tert-butoxide (1.68 g, 15.0 mmol) were added to toluene (15 ml) under N_2. The reaction mixture was stirred at reflux for 16 h. The reaction mixture was cooled to room temperature. Purification by silica column chromatography (SiO_2: hexanes/EtOAc, 10:1) yielded 1.55 g (44%) of yellow/brown solid product. ^{1}H NMR (400 MHz,

$CDCl_3$) δ = 7.20 (d, J = 8.6 Hz, 4H), 6.94 (d, J = 8.6 Hz, 4H), 5.62 (br, 1H), 2.42 (s, 6H). ^{13}C NMR (101 MHz, $CDCl_3$) δ = 141.29, 129.91, 129.32, 118.60, 77.48, 77.16, 76.84, 17.90. HRMS (ESI) m/z: 262.0721 [(M+)] calcd for $C_{14}H_{16}NS_2$ m/z: 262.0724

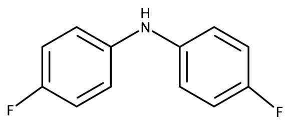

Figure S16: BPA-F

BPA-F (bis(4-fluorophenyl)amine): 4-bromo-fluorobenzene (3.15 g, 18 mmol), 4-fluoroaniline (2.00 g, 18 mmol), palladium acetate (0.29 g, 1.4 mmol), tri-tert-butylphosphine (0.32 g, 1.4 mmol) and potassium tert-butoxide (2.00 g, 90 mmol) were added to toluene (15 ml) under N2. The reaction mixture was stirred at reflux for 16 h. The reaction mixture was cooled to room temperature. Purification by silica column chromatography (SiO_2: hexanes/EtOAc, 5:1) yielded 2.29 g (62%) of dark brown oil product. ^1H NMR (400 mHz, $CDCl_3$) δ = 6.97, ((d, J = 1.1 Hz, 8 H), 5.46 (s, 1H). ^{13}C NMR (100 MHz, Acetone-d6): δ = 159.11, 156.73, 139.95, 119.59, 119.51, 116.23, 116.00 ppm. HRMS (EI) m/z: 205.07029 [(M+)] calcd for $C_12H_9NF_2$ m/z: 205.07031.

"spiro-OMe" (N2,N2,N2',N2',N7,N7,N7',N7'-octakis(4-methoxyphenyl)spiro[fluorene-9,9'-xanthene]-2,2',7,7'-tetraamine): bis(4-methoxyphenyl)amine (1.14 g, 5.00 mmol), HTM-Br_4 (0.678 g, 1.00 mmol), palladium acetate (0.018 g, 0.08 mmol), tri-tert-butylphosphine (0.018 g, 0.08 mmol) and potassium tert-butoxide (0.45 g, 5.00 mmol) were added to toluene (15 ml) under N2. The reaction mixture was stirred at reflux for 16 h. The reaction mixture was cooled to room temperature. Purification by silica column chromatography (SiO_2: hexanes/EtOAc, 4:1) yielded 0.731 g (59%) of product as pale green solid. 1H NMR (400 mHz, $CDCl_3$) δ = matched literature. 13C NMR (100 MHz, $CDCl_3$): δ = 155.68, 155.44, 154.94, 147.75, 146.84, 143.32, 141.73, 141.41, 133.14, 125.60, 125.51, 124.42, 124.15, 122.81, 121.88, 119.77, 118.73, 117.43, 114.65, 114.53. 55.61, 54.45. HRMS (ESI) m/z: 1241.5085 [(M+H)] calcd for C81H69N4O9 m/z: 1241.5065.

"spiro-p,o-OMe" (N2,N2',N7,N7'-tetrakis(2-methoxyphenyl)-N2,N2',N7,N7'-tetrakis(4-methoxyphenyl) spiro[fluorene-9,9'-xanthene]-2,2',7,7'-tetraamine):

2-methoxy-N-(4-methoxyphenyl)aniline (1.27 g, 5.50 mmol), HTM-Br_4 (0.800 g, 1.23 mmol), palladium acetate (0.020 g, 0.1 mmol), tri-tert-butylphosphine (0.022 g, 0.1 mmol) and potassium

tert-butoxide (0.50 g, 5.50 mmol) were added to toluene (15 ml) under N2. The reaction mixture was stirred at reflux for 16 h. The reaction mixture was cooled to room temperature. Purification by silica column chromatography (SiO_2: hexanes/EtOAc, 4:1) yielded 0.686 g (45%) of product as pale green solid. 1H NMR (400 mHz, CD2Cl2) δ = 7.24 (d, J = 9.0 Hz, 2H), 7.12 (m, 4H), 8.97 (m, 4H), 6.85 (m, 14H), 6.71 (m, 18H), 6.16 (d, J = 2.7 Hz, 2H), 3.73 (d, J = 15.4 Hz, 12H), 3.46 (d, J = 12.9 Hz, 12H) . 13C NMR (100 MHz, CD2Cl2): δ = 155.68, 155.57, 155.46, 154.95, 154.70, 147.67, 146.46, 143.17, 141.52, 140.95, 135.86, 135.84, 132.42, 129.26, 128.95, 126.23, 126.06, 125.89, 124.84, 122.85, 121.46, 121.35, 121.31, 119.74, 119.32, 119.08, 116.64, 116.29, 114.28, 114.22, 113.31, 113.11, 55.71, 55.60, 55.51, 55.50, 54.81. HRMS (ESI) m/z: 1240.4989 [(M+H)] calcd for C81H68N4O9 m/z: 1240.4986.

"spiro-Me" (N2,N2,N2',N2',N7,N7,N7',N7'-octa-p-tolylspiro[fluorene-9,9'-xanthene]-2,2',7,7'-tetraamine): di-p-tolylamine (1.46 g, 7.4 mmol), HTM-Br_4 (1.00 g, 1.5 mmol), palladium acetate (0.024 g, 0.12 mmol), tri-tert-butylphosphine (0.027 g, 0.12 mmol) and potassium tert-butoxide (0.71 g, 5.00 mmol) were added to toluene (15 ml) under N2. The reaction mixture was stirred at reflux for 16 h. The reaction mixture was cooled to room temperature. Purification by silica column chromatography (SiO_2: hexanes/EtOAc, 15:1) yielded 0.910 g (22%) of product as light brown solid. 1H NMR (400 mHz, Acetone-d8) δ = 7.46 (d, J = 8.3 Hz, 2H), 7.09-6.99 (m, 20H), 6.86 (d, J = 8.4 Hz, 8H), 6.82 (dd, J = 8.3, 2.3 Hz, 4H), 6.76 (d, J = 8.4 Hz, 8H), 6.39 (d, J = 2.6 Hz, 2H), 2.28 (s, 12H), 2.24 (s, 12H). 13C NMR (100 MHz, $CDCl_3$): δ = 156.03, 147.40, 147.36, 145.62, 145.50, 142.73, 133.87, 132.04, 131.29, 129.85, 129.69, 125.90, 125.30, 124.67, 123.89, 123.56, 122.65, 120.32, 120.13, 117.73, 77.47, 77.15, 76.84, 54.31, 20.94. HRMS (ESI) m/z: 1113.5469 [(M+H)] calcd for C81H69N4O m/z: 1113.5471.

"spiro-SMe": (N2,N2,N2',N2',N7,N7,N7',N7'-octa-p-thiomethylspiro[fluorene-9,9'-xanthene]-2,2',7,7'-tetraamine): bis(4-(methylthio)phenylamine (0.915 g, 3.5 mmol), HTM-Br_4 (0.454 g, 0.70 mmol), palladium acetate (0.040 g, 0.18 mmol), tri-tert-butylphosphine (0.036 g, 0.18 mmol) and potassium tert-butoxide (0.561 g, 5.00 mmol) were added to toluene (15 ml) under N2. The reaction mixture was stirred at reflux for 16 h. The reaction mixture was cooled to room temperature. Purification by silica column chromatography (SiO_2: hexanes/EtOAc, 12:1) yielded 0.451 g (47%) of product as yellow solid. 1H NMR (400 MHz, $CDCl_3$) δ 7.38 (d, J = 8.2 Hz, 2H), 7.39 -

7.09 (m, 16H), 7.02 (d, J = 8.8 Hz, 2H), , 6.93 - 6.79 (m, 22H), 6.36 (d, J = 2.6 Hz, 2H), 2.46 (s, 12H), 2.42 (s, 12H). 13C NMR (101 MHz, $CDCl_3$): δ

"spiro-FOMe"(N2,N2',N7',N7-tetrakis(4-fluorophenyl)-N2,N2',N7',N7-tetrakis(4-methoxyphenyl)spiro[fluorene-9,9'-xanthene]-2,2',7',7-tetraamine): N-(4-fluorophenyl)4-methoxyanaline (0.760 g, 3.5 mmol), HTM-Br_4 (0.454 g, 0.70 mmol), palladium acetate (0.18 g, 0.18 mmol), tri-tert-butylphosphine (0.036 g, 0.18 mmol) and potassium tert-butoxide (0.561 g, 5.0 mmol) were added to toluene (15 ml) under N2. The reaction mixture was stirred at reflux for 16 h. The reaction mixture was cooled to room temperature. Purification by silica column chromatography (SiO_2: hexanes/EtOAc, 20:1 to 10:1) yielded 0.620 g (74.2%) of product as pale brown solid. 1H NMR (400 MHz, $CDCl_3$) δ = 7.33 (d, 2H, J = 8.0 Hz), 6.98 (d, 2H, J = 8.0 Hz), 6.93- 6.76 (m, 38H), 6.33 (d, 2H, J = 2.7 Hz), 3.80 (s, 6H), 3.76 (s, 6H). 13C NMR (101 MHz, $CDCl_3$): δ 159.56, 159.26 (d, J = 60.6 Hz), 158.96, 156.86 (d, J = 59.3 Hz), 155.99, 155.69, 155.61, 147.59, 147.21, 144.63 (d, J = 2.5 Hz), 144.25 (d, J = 2.7 Hz), 143.08, 140.98, 140.82, 133.50, 126.17, 125.56, 125.51, 124.85, 124.70 (d, J = 7.8 Hz), 123.22, 122.97 (d, J = 7.7 Hz), 122.64, 120.13, 119.24, 117.78, 115.93 (d, J = 22.5 Hz), 115.68 (d, J = 22.4 Hz), 114.84, 114.74, 55.58, 55.55, 54.43.

"spiro-H"(N2,N2,N2',N2',N7,N7,N7',N7'-octaphenylspiro[fluorene-9,9'-xanthene]-2,2',7,7'-tetraamine): HRMS (ESI) m/z: 1001.4203 [(M+Na)] calcd for C71H54N4ONa m/z: 1001.4195. (Maciejczyk, J. Mater. Chem. A. 2016)

"spiro-F"(N2,N2,N2',N2',N7,N7,N7',N7'-octakis(4-fluorophenyl)spiro[fluorene-9,9'-xanthene]-2,2',7,7'-tetraamine): bis(4-fluorophenyl)amine (0.414 g, 2.0 mmol), HTM-Br_4 (0.300 g, 0.45 mmol), palladium acetate (0.012 g, 0.072 mmol), tri-tert-butylphosphine (0.018 g, 0.072 mmol) and potassium tert-butoxide (0.100 g, 0.87 mmol) were added to toluene (15 ml) under N2. The reaction mixture was stirred at reflux for 16 h. The reaction mixture was cooled to room temperature. Purification by silica column chromatography (SiO_2: hexanes/EtOAc, 15:1) yielded 0.204 g (39%) of product as light brown solid. 1H NMR (400 mHz, $CDCl_3$) δ = 7.36 (d, J = 8.8 Hz, 2H), 7.01 (d, J = 8.8 Hz, 2H, 6.89-6.77 (m, 38H), 6.26 (d, J = 2.6 Hz, 2H). 13C NMR (101 MHz, Chloroform-d) δ 160.03, 159.59, 157.61, 157.19, 155.49, 147.53, 147.44, 144.03, 144.01, 143.76, 142.91, 133.82, 129.16, 128.35, 125.57, 125.46, 125.38, 125.28, 124.35, 124.27, 123.48, 123.21, 120.41, 119.66, 118.06,

116.33, 116.12, 115.90, 54.45. HRMS (ESI) m/z: 1145.3438 [(M+Na)] calcd for C71H46N4OF8Na m/z: 1145.3442.

S5 Packing Density Analysis from Bulk Simulation

Table S4: Calculated Packing Densities from Quantum Patch Simulations for **Spiro-OMeTAD** and **Spiro-R** series

Compound	Packing Density (molecules nm^{-3})
**spiro-OMeTAD**	0.507499
**spiro-OMe**	0.474241
**spiro-*p*-,*o*-OMe**	0.505309
**spiro-Me**	0.527967
**spiro-SMe**	0.423663
**spiro-FOMe**	0.547382
**spiro-H**	0.621312
**spiro-F**	0.610558

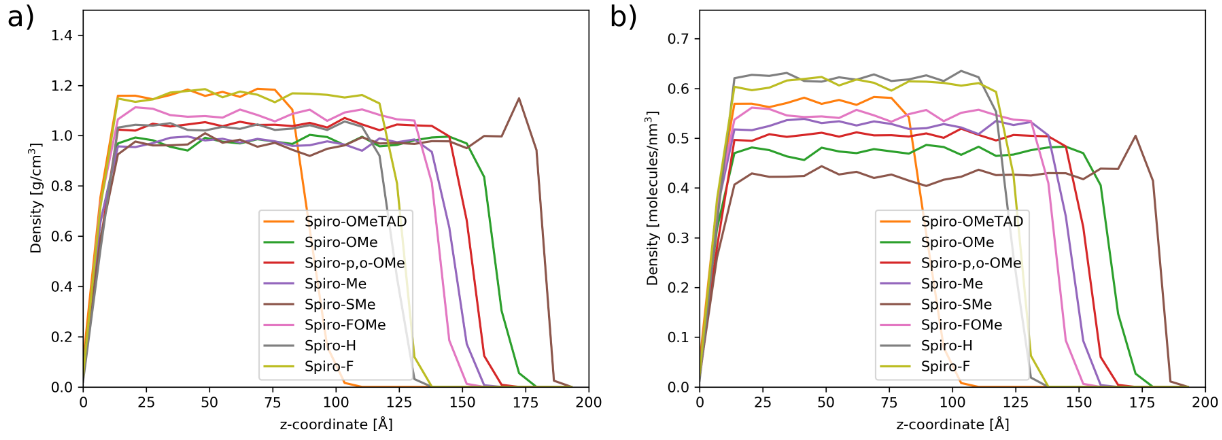

Figure S17: Simulated density profiles by both (a) mass and (b) molecular density.

S6 Correlation Plots Between Experimental and Simulated Values

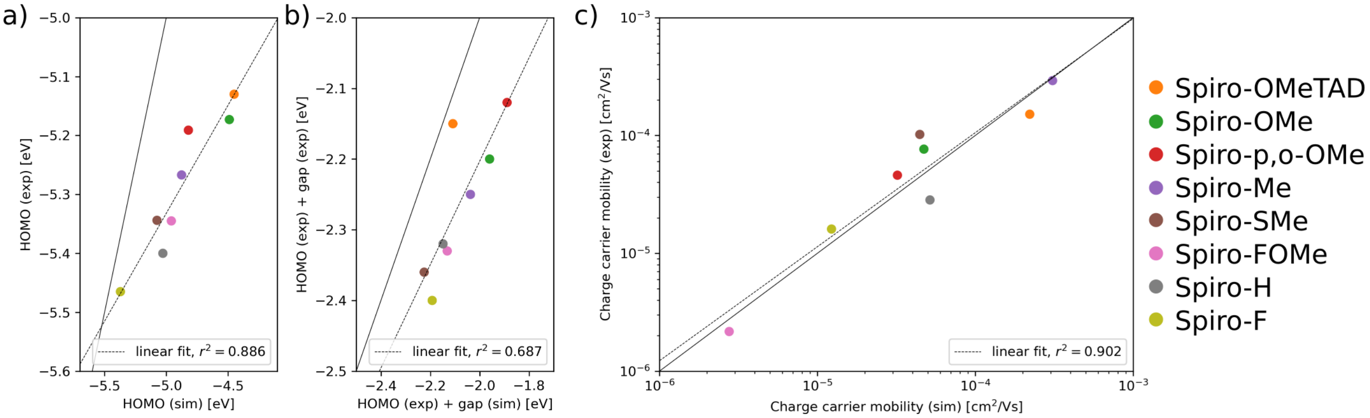

Figure S18: a) Correlation between experimental HOMO energy simulated E_{HOMO}.
b) Correlation between experimental HOMO energy and observed gap and simulated experimental HOMO energy and simulated HOMO-LUMO gap (LUMO energy).
c) Correlation between experimentally observed charge carrier mobility and simulated charge carrier mobility. All simulated values are obtained from the Quantum Patch Method simulations.

S7 Calculated Frontier Molecular Orbital Properties Based on Functional Groups

A table of all theoretical data is provided (screened_molecules_homo_lumo_energies.csv). The molecules are represented as SMILES strings in the column "smiles." These strings can be directly copy/pasted into chemical drawing programs, such as ChemDraw or MarvinSketch to visualize the molecules.

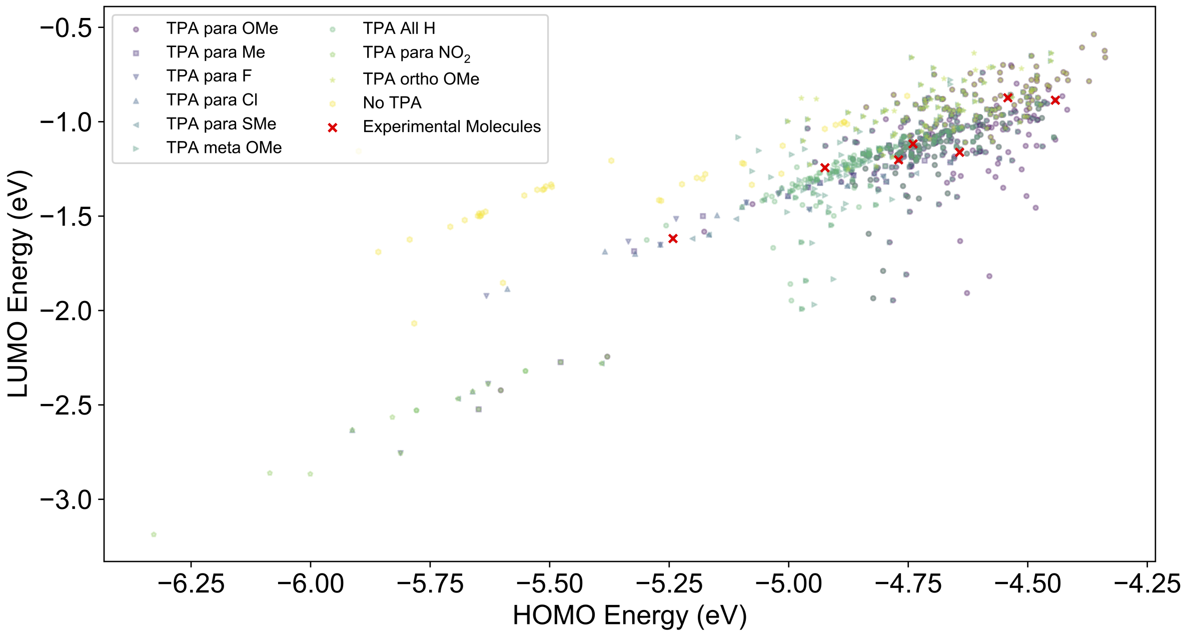

Figure S19: Plot of LUMO and HOMO (B3LYP/def2-SV(P)) as a function of functional group presence. No TPA means that functional groups are attached directly to the fluorene, xanthene, or biothiophene core. Since the functional groups are mixed in the screening library, each point can have multiple other coincident points.

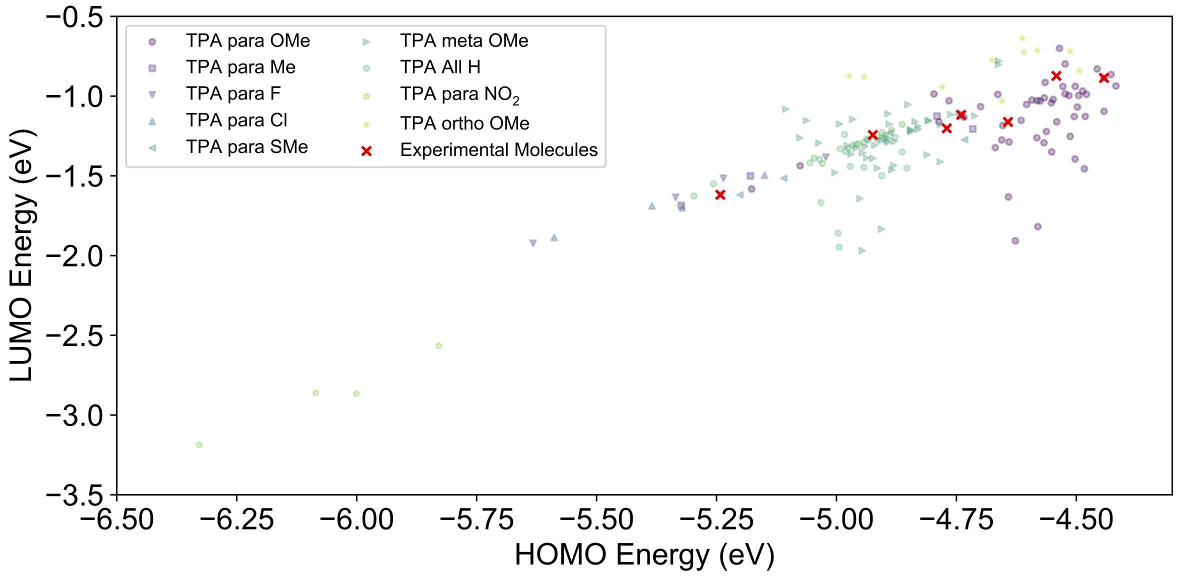

Figure S20: Plots of LUMO and HOMO (B3LYP/def2-SV(P)) for molecules with a single type of functional group, with the experimentally selected molecules (some of which have mixed functional groups) superimposed.

S8 Frontier Molecular Orbital Energies in Vacuum and in Implicit Solvent

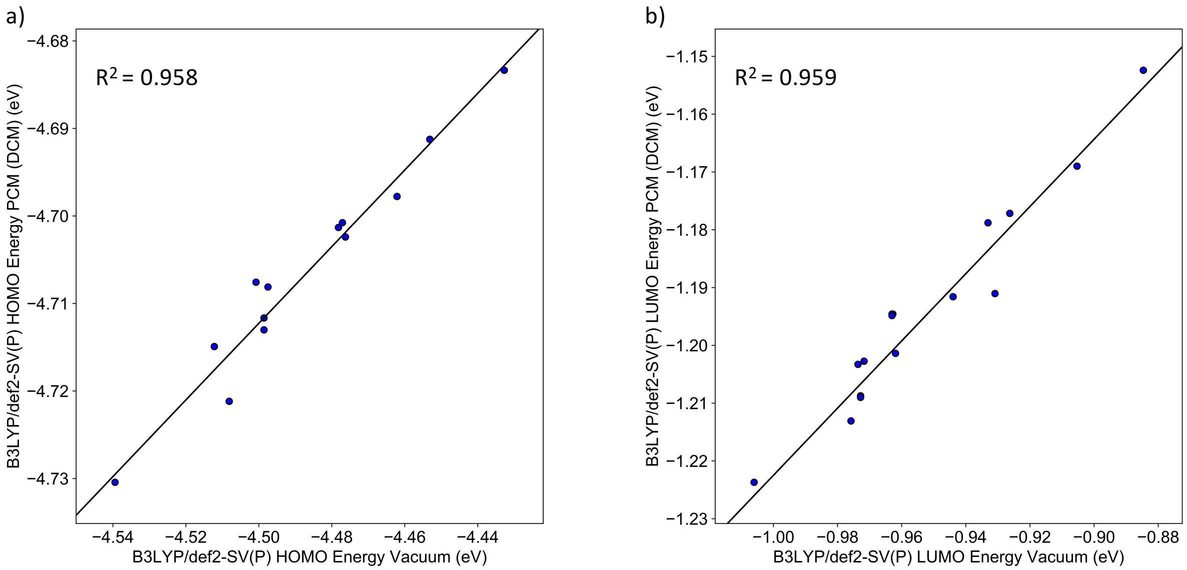

Figure S21: Plots of correlation between HOMO and LUMO (B3LYP/def2-SV(P)) for conformers of the **spiro-R** series and **spiro-OMeTAD** in vacuum and in a PCM implicit solvent (with DCM as the implicit solvent). Though there is a non-unity slope in this plot, for this subset of the data, there is a strong correlation between the vacuum and the PCM calculation when a linear model is applied. One could screen at the PCM level after conducting the vacuum calculation, though the computational cost is higher with molecules of this size.
